# Beneficial Effects of Anti-Interleukin-6 Antibodies on Impaired Gastrointestinal Motility, Inflammation and Increased Colonic Permeability in a Murine Model of Sepsis Are Most Pronounced When Administered in a Preventive Setup

**DOI:** 10.1371/journal.pone.0152914

**Published:** 2016-04-04

**Authors:** Sara Nullens, Michael Staessens, Cédric Peleman, Philip Plaeke, Surbhi Malhotra-Kumar, Sven Francque, Joris G. De Man, Benedicte Y. De Winter

**Affiliations:** 1 Laboratory of Experimental Medicine and Pediatrics, Division of Gastroenterology, University of Antwerp, Antwerp, Belgium; 2 Laboratory of Medical Microbiology, University of Antwerp, Antwerp, Belgium; 3 Antwerp University Hospital, Department of Gastroenterology and Hepatology, Antwerp, Belgium; University of Leicester, UNITED KINGDOM

## Abstract

**Background and Objectives:**

During sepsis, gastrointestinal ileus, mucosal barrier dysfunction and bacterial translocation are accepted to be important triggers that can maintain or exacerbate the septic state. In the caecal ligation and puncture animal model of sepsis, we demonstrated that systemic and colonic interleukin-6 levels are significantly increased coinciding with an impaired colonic barrier function. We therefore aimed to study the effect of therapeutic or curative administration of anti-IL6 antibodies on overall GI motility, colonic permeability and translocation of intestinal bacteria in blood and mesenteric lymph nodes in the mouse caecal ligation and puncture model.

**Methods:**

OF-1 mice were randomized to either the preventive or curative protocol, in which they received 1 mg/kg of antibodies to interleukin-6, or its IgG isotype control solution. They subsequently underwent either the caecal ligation and puncture procedure, or sham-surgery. GI motility was assessed 48h following the procedure, as well as colonic permeability, serum and colon cytokines, colonic tight junction proteins at the mRNA level; cultures of blood and mesenteric lymph nodes were performed.

**Results:**

Preventive administration of anti-interleukin-6 antibodies successfully counteracted the gastrointestinal motility disturbances and impaired colonic barrier function that could be observed in vehicle-treated septic animals. Serum and colonic levels of proinflammatory cytokines were significantly lower when animals were preventively treated with anti-interleukin-6 antibodies. A repetitive injection 24h later resulted in the most pronounced effects. Curative treatment significantly lowered systemic and colonic inflammation markers while the effects on transit and permeability were unfortunately no longer significant.

**Conclusions:**

Caecal ligation and puncture resulted in septic ileus with an increased colonic permeability. Antibodies to interleukin-6 were able to ameliorate gastro-intestinal motility, suppress inflammation and normalize the permeability of the colonic wall, with the preventive administration combined with a repeat injection being far more efficacious than the sole preventive or curative one.

## Introduction

Sepsis can be defined as a diverse clinical entity that ranges from the mere presence of bacteria in the blood stream to the development of a multiple organ dysfunction syndrome, with mortality rates ranging somewhere between 25 and 50% depending on the severity and presence of hemodynamic shock [1,2]. The gastrointestinal tract (GI tract) is nowadays regarded to play an important role in sepsis with the development of paralytic ileus, defined as an inhibition of the propulsive motility of the entire GI tract, and failure of the mucosal barrier function [3]. In the *2013 Surviving Sepsis Campaign*, the most recent guidelines on the approach of the septic patient, actively targeting the GI tract is still a matter of debate: the authors suggest that selective oral and digestive tract decontamination by means of antibiotics should be investigated within the scope of infection prevention and that further research is warranted in order to ascertain its benefits, risks and cost-effectiveness [4]. The GI tract is by many considered to play a major role in the development and maintenance of sustained inflammation and organ failure during sepsis [5]. Recent animal experiments showed that gut-derived factors in lymph collected from mesenteric lymph nodes can damage distant organ systems in an animal model of trauma followed by hemorrhagic shock [6], supporting the ‘gut-lymph’ hypothesis and the importance of the failing GI tract during sepsis [5,7].

A major proinflammatory cytokine that plays a pivotal role in the pathogenesis of sepsis is interleukin-6 (IL-6). Its major detrimental proinflammatory effects are mediated via the binding of IL-6 to its soluble receptor (sIL-6R). This IL-6 –sIL-6R complex will subsequently bind onto the signaling receptor protein gp130 (gp130), which is ubiquitously expressed on all cells, a process termed *trans-signaling*. Its effects are extensively reviewed elsewhere [8,9]. *Trans-signaling* is the major pathway via which the immune system is activated, and will play an important role in the transition from innate towards acquired immunity, the release of acute phase reagents, the secretion of immunoglobulins from B cells and the skewing of T cells towards a predominantly Th17 subtype in favor of the regulatory T cell subset [10,11]. *Classic signaling* occurs when IL-6 binds onto a membrane-bound IL-6R, and is mainly responsible for its anti-inflammatory and regenerative effects [8].

Besides its pro- and anti-inflammatory properties, it was already acknowledged decades ago that IL-6 has a detrimental effect on several epithelial layers [12–14]. The presence of IL-6 is mandatory for the development of gut barrier dysfunction, as Yang *et al* showed that IL-6 KO mice were protected from developing subsequent impaired GI barrier function in a mouse model of hemorrhagic shock [15]. However, IL-6 KO mice displayed more severe inflammation in a mouse model of DSS-colitis, which can be explained by the absence of the regenerative effects of IL-6 on intestinal epithelial cells [16,17].

In the search for a new therapeutic target, anti-cytokine strategies are dealing with a somewhat bad reputation in the field of sepsis. The translation from murine studies towards the human medical treatment was often unsuccessful [18,19]). Murine sepsis models have provided us with an extensive knowledge on the mechanisms of sepsis, but these experimental models are often performed under tightly controlled circumstances, making extrapolation towards the ‘average’ septic patient population nearly impossible [18–21]. Many authors however postulate that stratifying patients based upon their immunological profile prior to interventions targeting the immune system, may yield more beneficial results. Currently, trials targeting IL-6 remain, however, limited to pathologies such as rheumatoid arthritis, polymyalgia and various forms of cancer.

In previous research we observed a significant increase in serum and colonic levels of IL-6 in the caecal ligation and puncture (CLP) model, that coincided with a disturbance of the colonic barrier function [22]. So far conflicting data on the blockage of IL-6 in animal models of sepsis were reported (S1 Table*)*. Briefly, some authors demonstrated an improvement in survival [23–27], whereas others failed to demonstrate a survival benefit [28]). This improved survival was furthermore demonstrated to be dependent on the amount of antibody that was infused [25,29]. Studies so far focused primarily on survival and provided little data on the pivotal role of the gastrointestinal tract during sepsis. Wang *et al* showed that septic IL-6 KO mice did not display increased GI permeability, whereas their septic wild-type counterparts displayed increased mucosal permeability [30]. We therefore aimed to further investigate the effects of directly blocking IL-6 on gastrointestinal inflammation, motility and permeability. Antibodies were administered either immediately prior to CLP (preventive setup) with or without a repeat-injection 24h following CLP, or in a curative fashion (only one injection 24h following CLP).

## Methods

### Mice

OF-1 mice, eight week old, were obtained from Charles River (France) and housed in groups of 6 animals in standardized conditions (12h light-dark cycle, 21 ± 1°C, 40–60% humidity) with unlimited access to regular chow and tapwater. Mice were allowed to acclimatize 10 days before the experiments. All experiments were approved by the Committee for Medical Ethics and the use of Experimental Animals at the University of Antwerp (file number 2012–42).

### Products purchased

The following products were used: Xylazine 2% (Rompun®), Bayer; ketamine 50 mg/mL (Ketalar®), Pfizer; buprenorphin 0.3 mg/mL (Temgesic®), Schering-Plough; diethyl ether, Chem-lab NV, Belgium; saline (0.9% NaCl), Braun; Cytometric Bead Array (CBA) Bead Based Assay for IL-6, TNF-α, IL-10, IL-17A, IL-1α and IL-1β, BD Biosciences, Belgium; *Evans blue* (dye content >75%) (EB) and SIGMA*FAST* protease inhibitor tablets, Sigma-Aldrich; Dulbecco’s Phosphate Buffered Saline (PBS), Gibco Life Technologies; rat anti-mouse IL-6 Functional Grade Purified antibodies (clone MP5-20F3) and rat IgG1 isotype control purified antibodies (clone eBRG1) from eBioscience.

### Caecal ligation and puncture model

**Fig 1:**
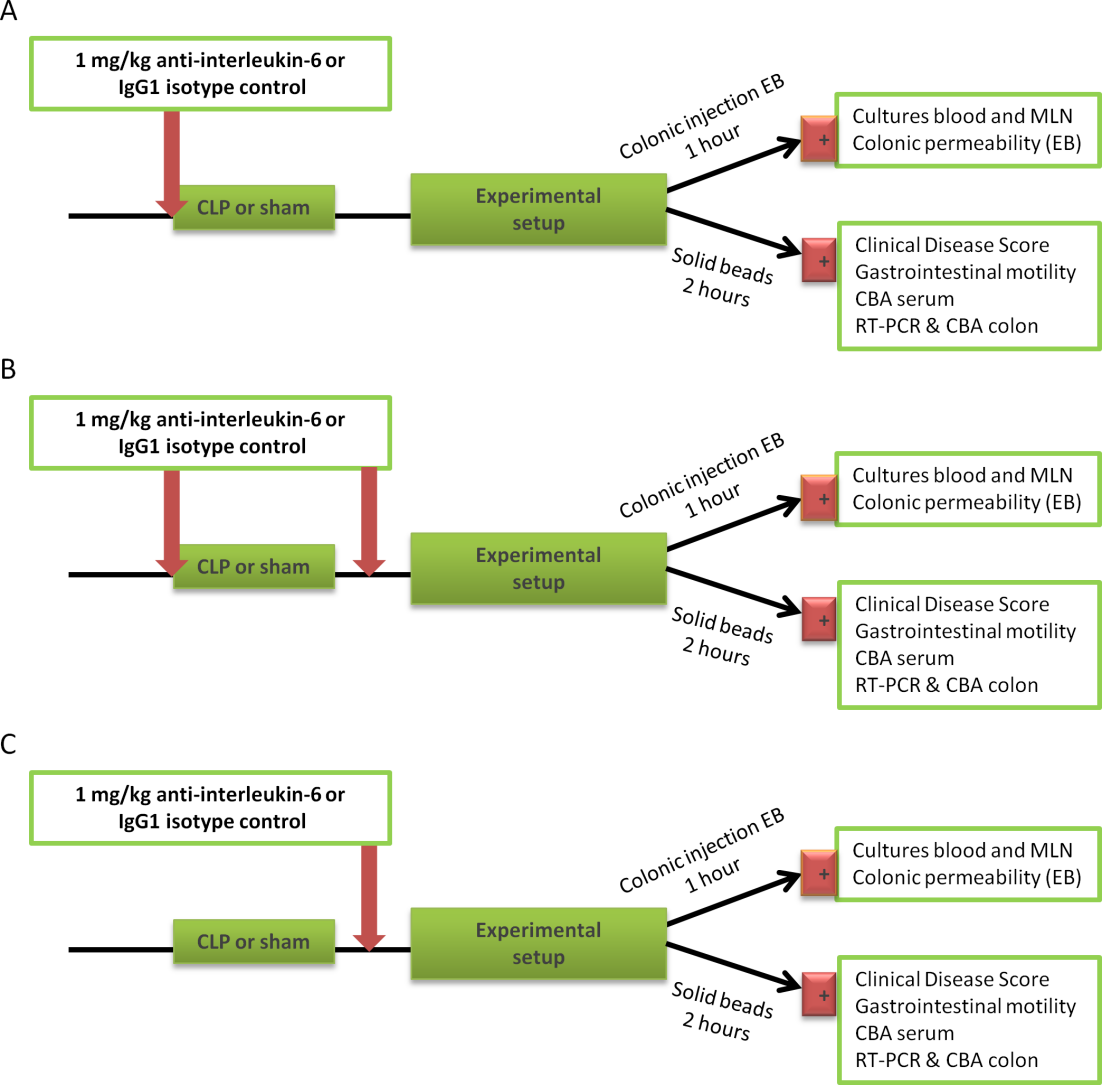
Experimental design of the study. In a first experimental setup (A), mice received anti-interleukin-6 antibodies (1 mg/kg) or the vehicle (IgG1 kappa isotype) simultaneously with the CLP- or sham-procedure. In a second setup (B), mice furthermore received a repeated injection with the antibodies 24h following the procedure. In a third setup (C), mice only received anti-IL-6 or vehicle 24h following the CLP- or sham-procedure. 48h post-CLP, mice received a gavage of colored solid beads and 2h later mice were sacrificed for measurement gastrointestinal transit, prelevation of serum and tissue samples, measurement of colonic permeability and quantification of cell adhesion molecules at the mRNA and protein level. CBA: cytometric bead array; CDS: clinical disease score; CLP: caecal ligation and puncture; MLN: mesenteric lymph nodes; mRNA: messenger ribonucleic acid; RT-PCR: reverse transcriptase real-time polymerase chain reaction.

In order to induce sepsis, the caecal ligation and puncture procedure was performed as previously described [20,22]. In short, mice were anesthetized with a mixture of ketamine (60 mg/kg i.p.) and xylazine (6.67 mg/kg i.p.) and placed onto a heating pad in the supine position. Following abdominal shaving and disinfection, a midline laparotomy was performed. The caecum was exteriorized and positioned onto moist sterile cottons, ligated for 50% with a 4/0 silk thread and punctured once through-and-through with a 25G needle in order to obtain a mild sepsis without mortality until sacrifice. The ligated caecum was repositioned into the abdominal cavity, and the abdomen was closed in layers with 5/0 Ethilon sutures. Mice received 1 mL of fluid resuscitation subcutaneously (s.c.) consisting out of a 37°C warm isotonic mixture of saline and glucose, combined with 0.05 mg/kg of buprenorphine s.c. for pain relief. Mice were allowed to recover in a heated cage (28°C) with free access to tapwater. Sham-operated mice received a midline incision without ligation or puncturing of the caecum. The experiments described below were initiated 48h following the CLP or sham-procedure (Fig 1). Animals were prematurely sacrificed when they lost over 15% of their baseline body weight or when they appeared to be moribund or had a clinical disease score (CDS) > 8 [31] (*Table 1*). Data from animals that were prematurely euthanized or had succumbed during the course of the experiment were not used in the final analysis.

**Table 1:** The *clinical disease score* (CDS) as applied in our study to score signs of disease in individual mice.

*Sign*	*Score*
Piloerection (not present–extensive)	0–2
Conjunctivitis (not present–bilateral)	0–2
Grooming behaviour (normal–none)	0–2
Mobility (normal–reduced–immobile)	0–3
Signs of peritoneal irritation (none–tiptoeing and broad pace)	0–2
Position of the ears (erect–flat)	0–1
Stool consistency (normal–sticky or diarrhea)	0–1
Anemic appearance (absent or present)	0–1
Moribund	0–1
Total *Clinical Disease Score* (no signs of disease–maximum)	0–15

### Experimental design

In a first set of experiments, a preventive protocol was designed in which mice received a single i.p. injection of anti-IL-6 antibodies (1 mg/kg) or its control compound (an irrelevant IgG1 kappa isotype control, 1 mg/kg) simultaneously with the CLP- or sham-procedure. Based upon data previously published in literature and own preliminary experiments (data not shown), 1 mg/kg was chosen as the optimal dose [25,29]. In a second set of experiments (preventive protocol with repeated injection), mice did additionally receive a second injection with the antibodies or the control compound 24h following the CLP-or sham-procedure. In a third set of experiments, a curative protocol was designed in which mice only received a single i.p. injection of anti-IL-6 antibodies (1 mg/kg) or its control solution (an irrelevant IgG1 kappa isotype control, 1 mg/kg) 24h following the CLP- or sham-procedure (Fig 1).

In each experimental protocol, a first group of animals (n = 7–10/group) was used to assess GI motility. Sickness behavior was quantified based upon the clinical disease score (CDS) shown in Table 1. Following terminal anaesthesia, animals were sacrificed by cardiac exsanguination while obtaining blood samples (Multivette® 600 capillary blood collection, Sarstedt). Blood samples were centrifuged (10000 g, 5 min, 20°C) and supernatants were stored at -80°C until further analysis. Inflammation was furthermore assessed locally at the colonic level (see methods below). In order to calculate the number of animals needed per group, power analysis was performed using G*Power version 3.1.7 based upon results obtained from preliminary and previously performed comparable experiments [22]).

In each experimental protocol, a second group of animals (n = 9–12/group) was implemented to assess colonic permeability in septic and control animals under terminal anesthesia 48h following the CLP- or sham-procedure. Blood and mesenteric lymph nodes were obtained from all animals for culturing experiments (see below) to study bacterial translocation.

### *In vivo* measurement of gastrointestinal transit: the solid beads method

Mice were overnight deprived of food with free access to water. A gavage was given 48h following sham or CLP with 0.5 mL of sterile water containing 25 glass green-colored beads (diameter 0.3 mm) through a 20G flexible catheter (Terumo; outer diameter 1.10 mm, inner diameter 0.80 mm). Mice were subsequently sacrificed 2h following the gavage and the GI tract was resected from the distal esophageal sphincter until the anal verge, and divided into 10 parts (stomach, 5 small bowel segments, caecum, proximal colon, distal colon and faeces). The number of beads in every segment was counted under a stereomicroscope for calculation of the percentage gastric emptying (%GE) and the geometric center of intestinal transit (GC) as a marker for GI transit [32].

### Systemic cytokines

The concentration of IL-6, TNF-α, IL-10, IL-17A, IL-1α and IL-1β in serum (pg/mL) was determined using the BD CBA Bead Based Immunoassay on an Accuri® flow cytometer (BD Biosciences) according to the manufacturer’s instructions. Data were processed using FCAP Array (BD).

### Local colonic cytokine concentrations and cell adhesion proteins

Colonic cytokine levels were determined at the protein as well as the mRNA level. For the protein concentrations, whole colons were rinsed with phosphate buffered saline, blotted dry, weighed and placed in ice-cold Tris-EDTA buffer (PBS with 10 mM Tris and 1 mM EDTA) containing the SigmaFAST protease inhibitor cocktail (100 mg colon per mL). Tissues were minced, homogenized and centrifuged (11000 g, 4°C, 10 min) and supernatants were collected and assessed for levels of the pro-and anti-inflammatory cytokines mentioned above (pg/100 mg colon) using the BD CBA Mouse Cytokine Kit.

To determine cytokine content at the mRNA level, total RNA was isolated from a snap-frozen piece of colonic tissue using the Qiagen RNeasy Mini Kit and purity confirmed using the Nanodrop ND-1000 Spectrophotometer. Total RNA was treated with DNase to obtain DNA-free RNA (Turbu DNase-free, Life Technologies) and converted to cDNA using the Transcriptor First Strand cDNA Synthesis Kit (Roche Applied Science). Quantitative real-time PCR was performed using the TaqMan® Universal PCR Master Mix (Life Technologies) and primers as mentioned in (S2 Table). Out of four housekeeping genes, 18s ribosomal RNA was determined to be the optimal one to which the expression of genes were normalized against. The PCR reaction was performed in a 25μL reaction [33,34], with the following amplification parameters: 50°C for 2 min, 95°C for 10 min, followed by 40 cycles of 95°C for 15 sec and 60°C for 1 min. Furthermore, we performed real-time RT-PCR for different proteins of the tight junction (occludin, zonulin-1, claudin-1), desmosome (desmoglein-2) and adherens junction (E-cadherin).

### Colonic permeability

Colonic permeability was quantified by means of the *Evans blue* (EB) method [35–37] with an azo dye (Evans blue) that crosses epithelia paracellularly. Briefly, septic and control animals were kept sober overnight, anaesthetized terminally the next morning with a mixture of ketamine and xylazine and placed in the supine position on a heating pad to undergo an abdominal incision. Following abdominal disinfection and using sterile utensils and gloves, the colon was visualized and ligated distally of the ileocaecal valve and proximally of the anal sphincter. 100μL of a 9% EB solution (w/v) dissolved in PBS was injected into the colon with a Myjector U-100 insulin syringe, and the abdomen was closed in layers. One hour following the injection mice were sacrificed, colons resected and rinsed thoroughly with PBS containing 6 mM of acetylcysteine to flush out residual luminal EB and mucus. Colons were blotted dry, weighed and incubated for 24h in formamide (50°C, 95% O_2_, 5% CO_2_) to extract the EB absorbed by the GI wall as a measurement for colonic permeability. The extracted amount of EB from the colon was determined spectrophotometrically by measuring emission at 610 nm and expressed as μg EB/100 mg colonic tissue.

### Quantification of bacterial translocation

To estimate bacterial translocation into the bloodstream, one drop of EDTA-treated full blood was obtained by cardiac puncture from the animals in which colonic permeability was studied, and plated onto a blood agar culture plate following enrichment and incubated at 37°C for 24h in ambient air supplied with 5% CO_2_. Additionally, mesenteric lymph nodes (MLN) were resected aseptically, suspended in sterile PBS (10 mg MLN/100 μL PBS) and mashed manually using a 10 mL syringe plunger through a 40 μm nylon cell strainer. Homogenized MLN were plated onto a blood agar culture following enrichment.

### Histology and immunohistochemistry

A full thickness segment (0.5x0.5 cm) was taken from the proximal colon immediately adjacent to the caecum. The segment was fixed for 24h in 4% formaldehyde and subsequently embedded in paraffin. Transverse sections (5 μm) were stained with hematoxylline and eosin. Inflammation was scored by assessing the presence and degree of inflammatory infiltrates, presence of goblet cells, architecture of the crypts and presence of mucosal erosion as previously published [31], resulting in a cumulative score ranging from 0 (minimum) to 13 (maximum).

### Statistical analysis

Data are presented as mean ± SEM, with ‘n’ representing the number of mice. Two-way ANOVA followed by one-way ANOVA with post hoc Student-Newman-Keuls (SNK) analysis was applied to compare the results of the motility parameters, cytokine levels, PCR and other data as appropriate. The non-parametric Mann-Whitney U test was used to compare ordinal data (CDS). Data were analyzed using SPSS version 20.0 (IBM, Chicago) and visualized using GraphPad Prism version 6.00.

## Results

### Effect of preventive administration of anti-IL-6 antibodies on CLP-induced septic ileus

**Fig 2:**
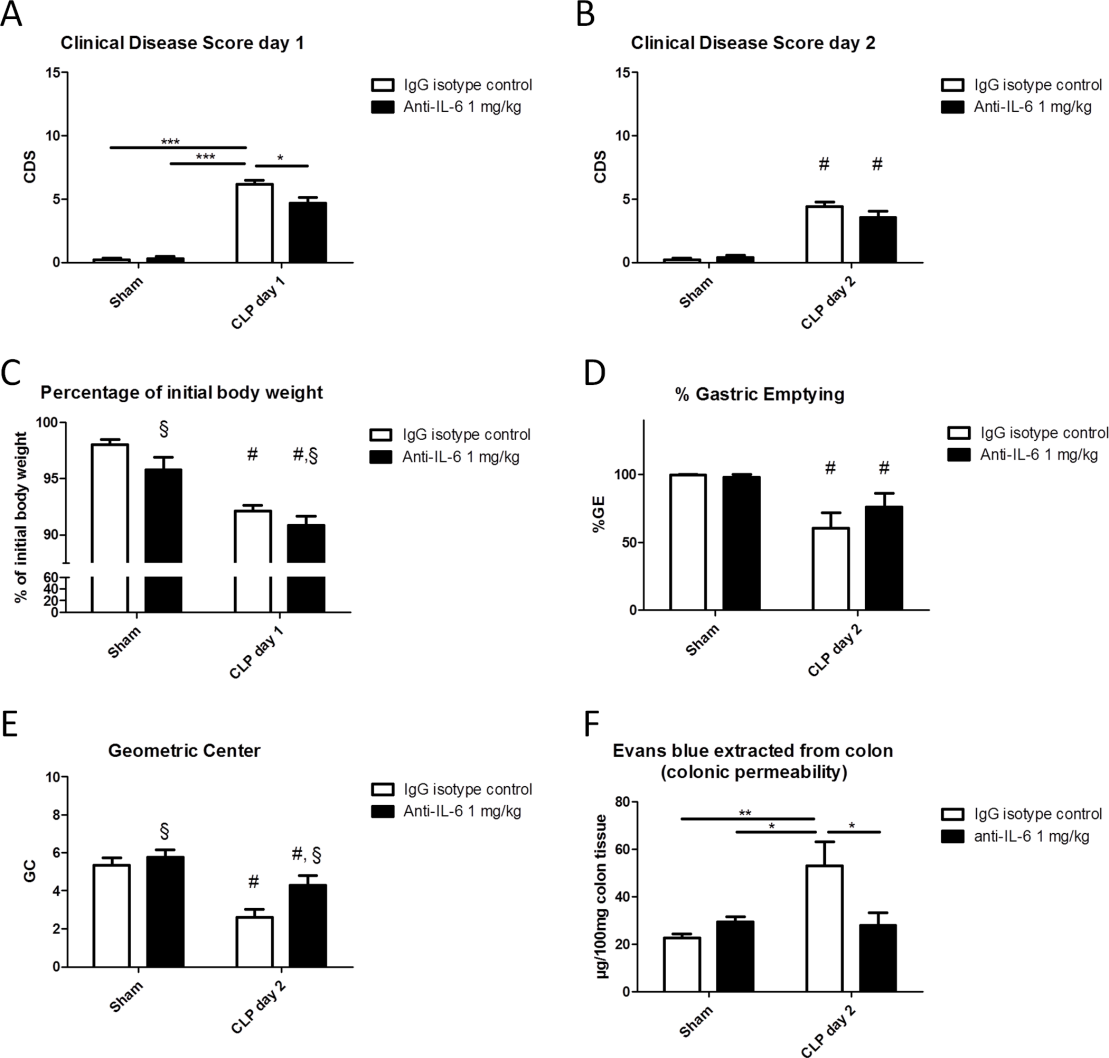
**Effect of** ***preventive*** **treatment with anti-IL-6 antibodies on sepsis-induced clinical signs of disease 24h (A) and 48h (B) following CLP or sham-surgery, percentage of weight loss at day 1 (C), percentage of gastric emptying (D), geometric center of GI transit (E) and colonic permeability as measured by the *Evans blue* method (F).** Two-way ANOVA followed by One-way ANOVA and SNK *post-hoc* testing when appropriate, or its non-parametric equivalent for ordinal data; n = 7–10/group for A, B and C; n = 9–12/group for D; *p ≤ 0.05, ***p ≤ 0/001, # significant effect of CLP, § significant effect of anti-IL-6. CDS: clinical disease score; CLP: caecal ligation and puncture; GC: geometric center; %GE: percentage of gastric emptying; GI: gastrointestinal.

CLP resulted in a reproducible occurrence of sepsis, and no difference in survival was noted between the anti-IL-6 treated and isotype-treated septic animal group as two animals in both groups had succumbed to the procedure prior to sacrifice. 24h following CLP, the CDS was significantly higher and body weight significantly lower in septic animals compared to sham-operated animals (Fig 2A and 2C). The septic animals that received anti-IL-6 antibodies demonstrated a significantly lower CDS. This beneficial effect of anti-IL-6 was not apparent anymore immediately prior to sacrifice 48h following CLP (Fig 2B). Sham-animals treated with anti-IL-6 antibodies did not display abnormal behavior or signs of illness.

CLP-induced sepsis resulted in an impairment of GI transit as represented by a significant drop in the intestinal geometric center. Administration of anti-IL-6 protected mice from developing an impaired GI transit, as demonstrated by a significantly greater intestinal geometric center (*Fig 2E*).

Following CLP-induced sepsis, we detected a significant rise in the serum levels of proinflammatory cytokines such as IL-6 and TNF-α, as well as a simultaneous increase in the serum levels of the anti-inflammatory IL-10 (Table 2). In septic mice, administering antibodies to IL-6 significantly decreased the concentration of serum IL-6 and IL-10. The decrease in TNF-α concentration did not reach significance. Similar to the serum results, the concentration of IL-6, TNF-α, IL-1β and IL-1α in the colonic wall rose significantly following CLP-induced sepsis (Table 2B). This rise in proinflammatory cytokine concentration was significantly reduced by anti-IL-6. These results were confirmed by the RT-PCR results on colonic tissue (Table 2C).

**Table 2:** Cytokine levels in serum (A) and supernatants of homogenized colons (B) measured by CBA or ELISA (IL-1α), and determined by RT-PCR in colon (C) during the *preventive* set-up (administration of anti-IL-6 antibodies simultaneously with the CLP- or sham-procedure).

	sham + IgG isotype	sham + anti-IL-6	CLP d2 + IgG isotype	CLP d2 + anti-IL-6
**A. Serum cytokines** (pg/mL)				
IL-6	4.56 ± 2.51	1.96 ± 0.55^§^	276.61 ± 71.64^#^	84.48 ± 18.30^#^^,^^§^
TNF-α	<L/D	2.28 ± 1.42	46.55 ± 11.86^#^	22.13 ± 7.56^#^
IL-10	<L/D	<L/D	12.43 ± 7.66	2.77 ± 1.45^a^
IL-1β	1.02 ± 0.52	1.25 ± 0.50	0.85 ± 0.44	1.65 ± 0.42
IL-17A	<L/D	<L/D	0.91 ± 0.26	0.89 ± 0.15
IL-1α	<L/D	<L/D	<L/D	<L/D
**B. Colon cytokines** (pg/100 mg colon tissue)				
IL-6	3.81 ± 2.38	2.18 ± 0.75	209.83 ± 94.59^#^	52.33 ± 14.06^#^
TNF-α	2.95 ± 1.94	0.45 ± 0.45	19.19 ± 9.27*	1.83 ± 1.20
IL-10	<L/D	<L/D	<L/D	<L/D
IL-1β	6.88 ± 2.27	5.10 ± 1.60	71.91 ± 54.84	8.01 ± 1.36
IL-17A	<L/D	<L/D	<L/D	<L/D
IL-1α	106.74 ± 17.45	111.05 ± 7.33	334.43 ± 82.70^#^	202.56 ± 33.93^#^
**C. mRNA colonic tissue**				
IL-6	1.73 ± 0.86	1.61 ± 0.15	136.18 ± 43.85^#^	70.18 ± 36.12^#^
TNF-α	1.52 ± 0.56	2.05 ± 0.60	4.91 ± 1.17^#^	3.22 ± 0.77^#^
IL-10	1.88 ± 0.80	3.11 ± 1.06	10.93 ± 4.46	4.00 ± 0.82
IL-1β	1.41 ± 0.42	2.40 ± 1.00	42.53 ± 20.77^#^	15.21 ± 7.21^#^

Two-way ANOVA followed by one-way ANOVA and SNK post-hoc testing when appropriate.

# significant effect of CLP

§ significant effect of anti-IL-6

For the SNK post-hoc testing

* p<0.05 as compared to the other three groups

^a^ <0.05 compared to CLP d2 + IgG isotype.

When sham concentrations were below the theoretical limit of detection, the unpaired Student’s t-test was subsequently applied to compare the CLP d2 + IgG isotype with the CLP d2 + anti-IL-6 group. For the PCR-results, data are expressed as relative expression (2^-ΔΔCT^ method) and the *sham + IgG isotype* group was chosen as calibrator. Data are presented as mean ± SEM. N = 8–12 animals per group.

<L/D, below the limit of detection; CBA, cytometric bead array; ELISA, enzyme-linked immunosorbent assay; IL, interleukin; TNF, tumor necrosis factor.

CLP significantly increased the colonic wall permeability to *Evans blue*. Preventive treatment with anti-IL-6 had no effect in controls but decreased the impaired permeability in septic mice (Fig 2F).

Blood cultures from sham mice were consistently negative, whereas 78% of blood samples from septic mice were positive. Anti-IL-6 antibodies did, however, not significantly reduce the number of positive blood cultures (Table 3). Treatment with anti-IL-6 also had no favorable effect on the outcomes of the MLN cultures, as these were 100% positive in treated as well as untreated septic animals.

**Table 3:** Cultures of blood and homogenized mesenteric lymph nodes.

	Sham + IgG isotype	sham + anti-IL-6	CLP d2 + IgG isotype	CLP d2 + anti-IL-6
**A. Preventive setup**				
Number of positive blood cultures (%)	0/6 (0%)	0/8 (0%)	7/9 (77.78%)*	5/9 (55.56%)*
Number of positive MLN cultures (%)	1/6 (16.67%)	2/8 (25%)	10/10 (100%)*	10/10 (100%)*
**B. Preventive setup + repeated injection**				
Number of positive blood cultures (%)	0/7 (0%)	1/6 (16.67%)	10/10 (100%)	8/11 (72.73%)
Number of positive MLN cultures (%)	0/7 (0%)	0/6 (0%)	10/10 (100%)	11/11 (100%)
**C. Curative setup**				
Number of positive blood cultures (%)	0/5 (0%)	0/5 (0%)	6/6 (100%)	6/6 (100%)
Number of positive MLN cultures (%)	0/5 (0%)	0/5 (0%)	6/6 (100%)	6/6 (100%)

Percentage of positive cultures from blood and homogenized mesenteric lymph nodes (24h incubation at 37°C, ambient air supplied with 5% CO_2_). Pearson’s chi-squared test for the percentage of positive cultures

* p ≤ 0.05 versus CLP day 2 + IgG isotype.

CFU: colony forming units; CLP: caecal ligation and puncture; MLN: mesenteric lymph nodes

The mRNA-levels of different cell adhesion proteins were all numerically upregulated during sepsis, with a significant peak in claudin-1, desmoglein-2 and E-cadherin mRNA levels. The administration of anti-IL-6 to septic animals induced an additional significant rise in the mRNA level of desmoglein-2, whereas claudin-1 levels were comparable to those in sham-operated animals (Table 4).

**Table 4:** RT-PCR of cell adhesion molecules.

	Sham + IgG isotype	sham + anti-IL-6	CLP d2 + vehicle	CLP d2 + anti-IL-6
**A. Preventive set-up**				
occludin	1.16 ± 0.26	1.97 ± 0.46	1.75 ± 0.26	1.88 ± 0.21
zonulin-1	1.17 ± 0.24	1.63 ± 0.29	1.56 ± 0.19	1.30 ± 0.14
claudin-1	1.44 ± 0.46	1.81 ± 0.81	3.04 ± 0.86*	2.21 ± 0.37
E-cadherin	1.11 ± 0.24	1.57 ± 0.22	2.19 ± 0.27^#^	1.82 ± 0.15^#^
desmoglein-2	1.19 ± 0.30	1.73 ± 0.43^1^	2.02 ± 0.29^#^	± 0.25^#^^,1^
**B. Preventive set-up + repeated injection**				
occludin	1.40 ± 0.58	1.05 ± 0.44^§^	1.82 ± 0.44	0.45 ± 0.05^§^
zonulin-1	1.01 ± 0.06	0.99 ± 0.20^2^	1.35 ± 0.08	0.98 ± 0.09^2^
claudin-1	1.10 ± 0.21	0.98 ± 0.30^§^	2.03 ± 0.67	0.49 ± 0.06^§^
E-cadherin	1.04 ± 0.11	0.92 ± 0.07^3^	1.45 ± 0.09^#^	1.22 ± 0.12^#^^,^^3^
desmoglein-2	1.05 ± 0.16	1.10 ± 0.33	2.70 ± 0.54*	± 0.09
**C. Curative set-up**				
occludin	1.36 ± 0.40	2.06 ± 0.34	1.96 ± 0.17*	0.96 ± 0.13
zonulin-1	1.48 ± 0.50	3.61 ± 0.53	3.46 ± 0.30	1.90 ± 0.25^a^
claudin-1	1.77 ± 0.76	2.56 ± 0.72	3.07 ± 0.46*	1.04 ± 0.24
E-cadherin	1.39 ± 0.42	2.34 ± 0.31	3.99 ± 0.31*	1.78 ± 0.24
desmoglein-2	1.57 ± 0.58	3.16 ± 0.53	3.95 ± 0.29	1.92 ± 0.28^a^

Two-way ANOVA followed by one-way ANOVA and SNK post-hoc testing when appropriate.

# significant effect of CLP

§ significant effect of anti-IL-6

* p < 0.05 compared to the other three groups

^a^ p < 0.05 as compared to CLP d2 + IgG isotype control and sham + anti-IL-6

^1^ p = 0.08 for the effect of anti-IL-6

^2^ p = 0.06 for the effect of anti-IL-6

^3^ p = 0.07 for the effect of anti-IL-6. N = 8–11 animals per group.

No obvious differences were observed on haematoxylin-eosin stain between control and septic animals, nor did preventive administration of anti-IL-6 result in any histological changes (score for all four groups: 0.0±0.0, p = NS). Therefore, the beneficial GI motility changes we observed following anti-IL-6 treatment in the septic animals were not associated with an amelioration of the GI histology (S1 Fig).

### Effect of preventive administration of anti-IL-6 antibodies with one repeated injection 24h later on CLP-induced septic ileus

**Fig 3:**
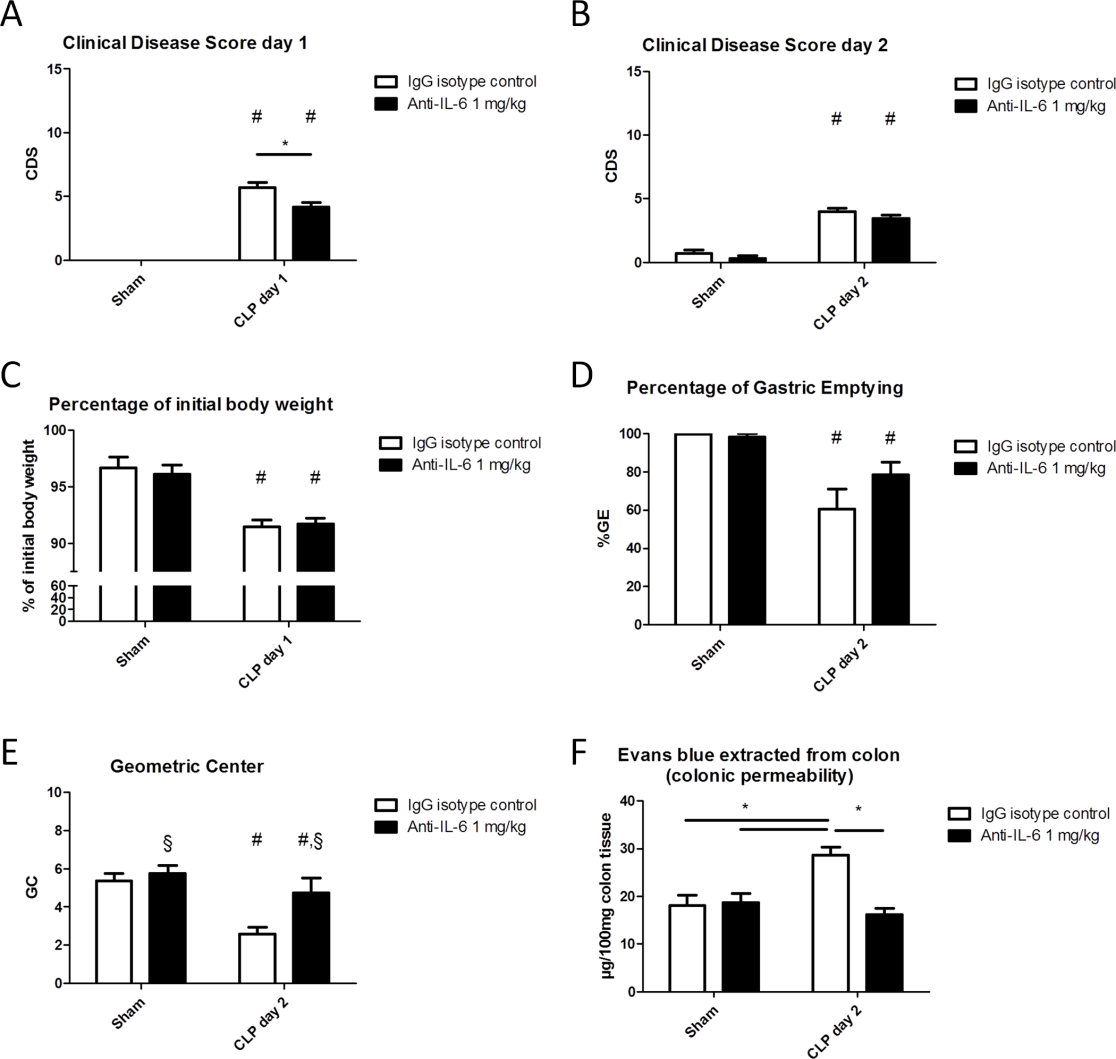
**Effect of preventive treatment and repeat injection with anti-IL-6 antibodies on sepsis-induced clinical signs of disease 24h (A) and 48 h (B) following CLP or sham-surgery, percentage of weight loss at day 1 (C), percentage of gastric emptying (D), geometric center of GI transit (E) and colonic permeability as measured by the Evans blue technique (F).** Two-way ANOVA followed by One-way ANOVA and SNK *post-hoc* testing when appropriate, or its non-parametric equivalent for ordinal data; n = 7–10/group for A, B and C; n = 9–12/group for D; *p ≤ 0.05, # significant effect of CLP, § significant effect of anti-IL-6. CDS: clinical disease score; CLP: caecal ligation and puncture; GC: geometric center; %GE: percentage of gastric emptying; GI: gastrointestinal.

**Fig 4:**
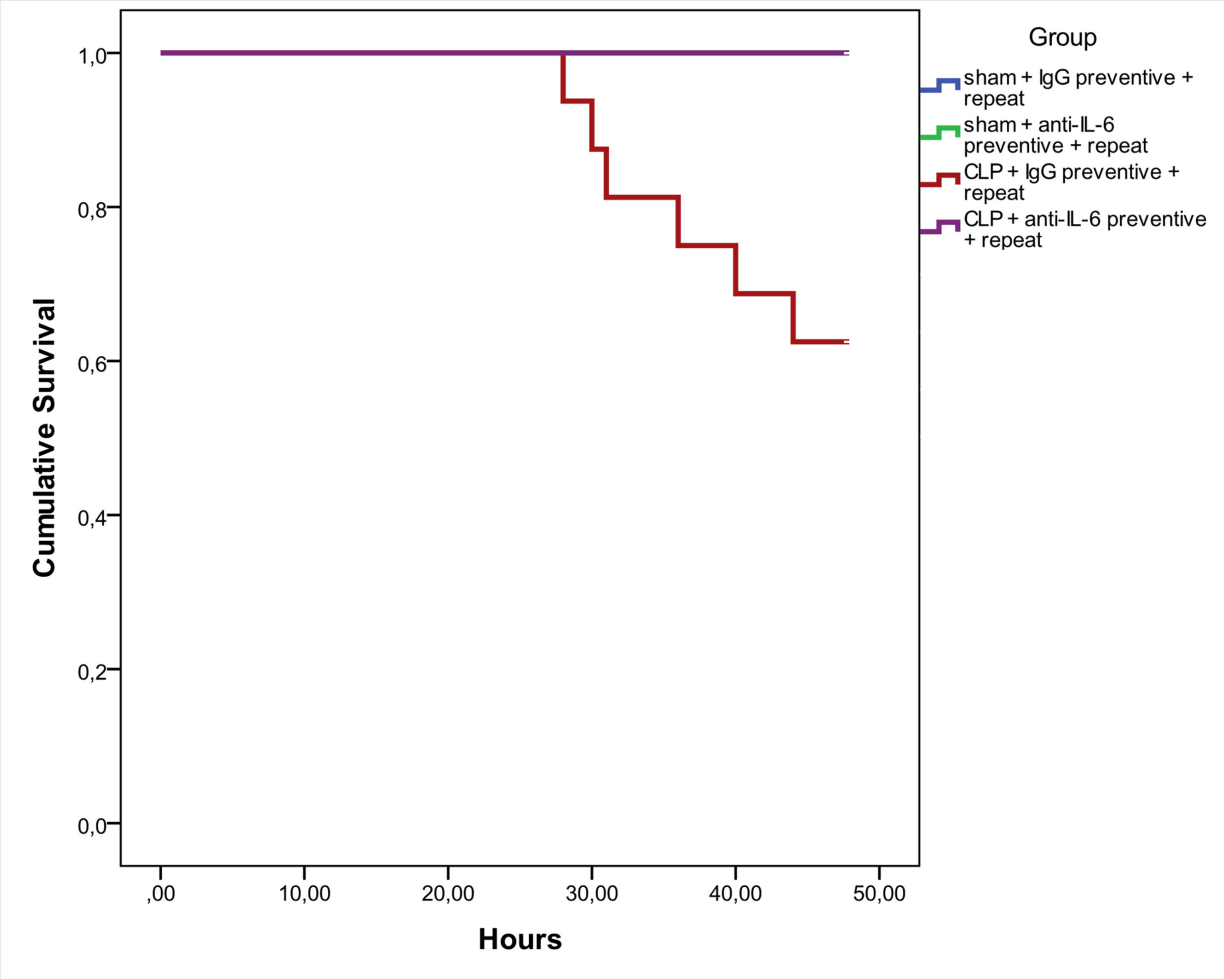
Kaplan-Meier survival analysis. Kaplan-Meier curve displaying survival of sham and CLP-mice treated with anti-IL-6 antibodies or IgG isotype control up until 48h following the CLP-procedure. Log rank test p = 0.011.

In a second experiment, animals received a second injection with antibodies specific to IL-6, or the IgG control compound. 24h following CLP, the CDS was significantly higher in animals subjected to CLP, whereas septic animals treated with anti-IL-6 antibodies displayed a significant drop in their CDS (Fig 3A). This effect on CDS was no longer apparent on day 2 prior to sacrifice (Fig 3B). The percentage of weight loss was significantly decreased in septic animals; however, no difference was noted between treated and untreated animals (Fig 3C). CLP-induced sepsis significantly impaired GI motility, as reflected by a drop in %GE and GC; repeated treatment with anti-IL-6 antibodies significantly increased the GC, whilst the effect on %GE was not statistically different (Fig 3D and 3E). Treatment with the antibodies normalized the colonic permeability in CLP-animals to that in sham animals, as can be deduced by the amount of Evans blue extracted from the colon (Fig 3F). Remarkably, a significant improvement in survival was observed in the septic animals treated twice with the antibodies, compared to the isotype-treated CLP-group (Fig 4).

Administering a second injection with anti-IL-6 antibodies to animals that were subjected to CLP normalized IL-6 levels in both serum and colon to that in control animals (Table 5A and 5B). Colonic IL-6 mRNA levels in septic animals however remained unaltered following treatment (Table 5C). TNF-α, IL-1β and IL-1α levels rose following CLP, but were not significantly altered after targeting interleukin-6. Remarkably, TNF-α levels paradoxically rose significantly in the colon as well as the serum following the repeated treatment with antibodies; we already observed this feature in previous research following the reduction of IL-6 to levels observed in control animals [22]. Blood cultures from control animals were consistently negative, whereas 100% of cultures from blood as well as MLN from untreated septic animals yielded positive results. Treating animals with anti-IL-6 levels however did not significantly influence the outcome of the cultures (Table 3).

**Table 5:** Cytokine levels in serum (A) and supernatants of homogenized colons (B) measured by CBA, and determined by RT-PCR in colon (C) during the *preventive* set-up with repeated injection (administration of anti-IL-6 antibodies simultaneously with the CLP- or sham-procedure, and repeated once 24h following the procedure).

	sham +2x isotype	sham +2x anti-IL-6	CLP d2 +2x isotype	CLP d2 + 2x anti-IL-6
**A. Serum cytokines** (pg/mL)				
IL-6	5.19 ± 2.42	0.68 ± 0.29	461.46 ± 99.66*	33.90 ± 3.12
TNF-α	2.96 ± 1.18	3.48 ± 0.34	39.56 ± 7.04^#^	48.89 ± 6.63^#^
IL-10	<L/D	<L/D	0.89 ± 0.89	2.03 ± 1.64
IL-1β	<L/D	<L/D	2.06 ± 1.90	1.25 ± 0.87
IL-17A	<L/D	<L/D	3.67 ± 0.83	1.10 ± 0.30
IL-1α	<L/D	<L/D	3.82 ± 2.40	2.75 ± 0.68
**B. Colon cytokines** (pg/100 mg colon tissue)				
IL-6	6.34 ± 1.29	3.73 ± 0.38	1049.23 ± 260.74*	69.11 ± 17.32
TNF-α	7.15 ± 2.81	7.71 ± 2.16	28.76 ± 4.89^#^	47.16 ± 10.88^#^
IL-10	<L/D	<L/D	<L/D	<L/D
IL-1β	1.75 ± 1.17	2.77 ± 1.36	28.26 ± 12.82^#^	36.63 ± 12.61^#^
IL-17A	<L/D	<L/D	1.61 ± 0.34	1.19 ± 0.46
IL-1α	1.38 ± 0.47	1.68 ± 0.30	21.15 ± 11.73^#^	20.37 ± 7.50^#^
**C. mRNA colonic tissue**				
IL-6	1.83 ± 0.86	1.19 ± 0.28	13.26 ± 2.85^#^	11.82 ± 2.05^#^
TNF-α	1.05 ± 0.13	1.12 ± 0.29	6.48 ± 1.51^#^	5.13 ± 0.64^#^
IL-10	1.07 ± 0.17	0.79 ± 0.13^§^	4.07 ± 0.77^#^	2.13 ± 0.28^#^^,^^§^
IL-1β	1.08 ± 0.18	0.85 ± 0.11^1^	6.86 ± 1.24^#^	4.09 ± 0.56^#^^,^^1^
IL-1α	1.16 ± 0.27	0.78 ± 0.07	5.69 ± 1.37^#^	4.64 ± 0.89^#^

Two-way ANOVA followed by one-way ANOVA and SNK post-hoc testing when appropriate.

# significant effect of CLP

§ significant effect of anti-IL-6

For the SNK post-hoc testing

* p<0.05 compared to the other three groups

^1^ p = 0.055 for the effect of anti-interleukin-6.

When sham concentrations were below the theoretical limit of detection, the unpaired Student’s t-test was subsequently applied to compare the CLP d2 with the CLP d2 + anti-IL-6 group. For the PCR-results, data are expressed as relative expression (2^-ΔΔCT^ method) and the *sham + IgG isotype* group was chosen as calibrator. Data are presented as mean ± SEM. N = 7–10 animals per group.

<L/D, below the limit of detection; CBA, cytometric bead array; ELISA, enzyme-linked immunosorbent assay; IL, interleukin; TNF, tumor necrosis factor.

The mRNa levels of E-cadherin and desmoglein-2 were significantly upregulated during in septic animals, and treatment with anti-IL-6 significantly decreased mRNA concentrations of occludin and claudin-1 (Table 4).

### Effect of curative administration of anti-IL-6 antibodies on CLP-induced septic ileus

**Fig 5:**
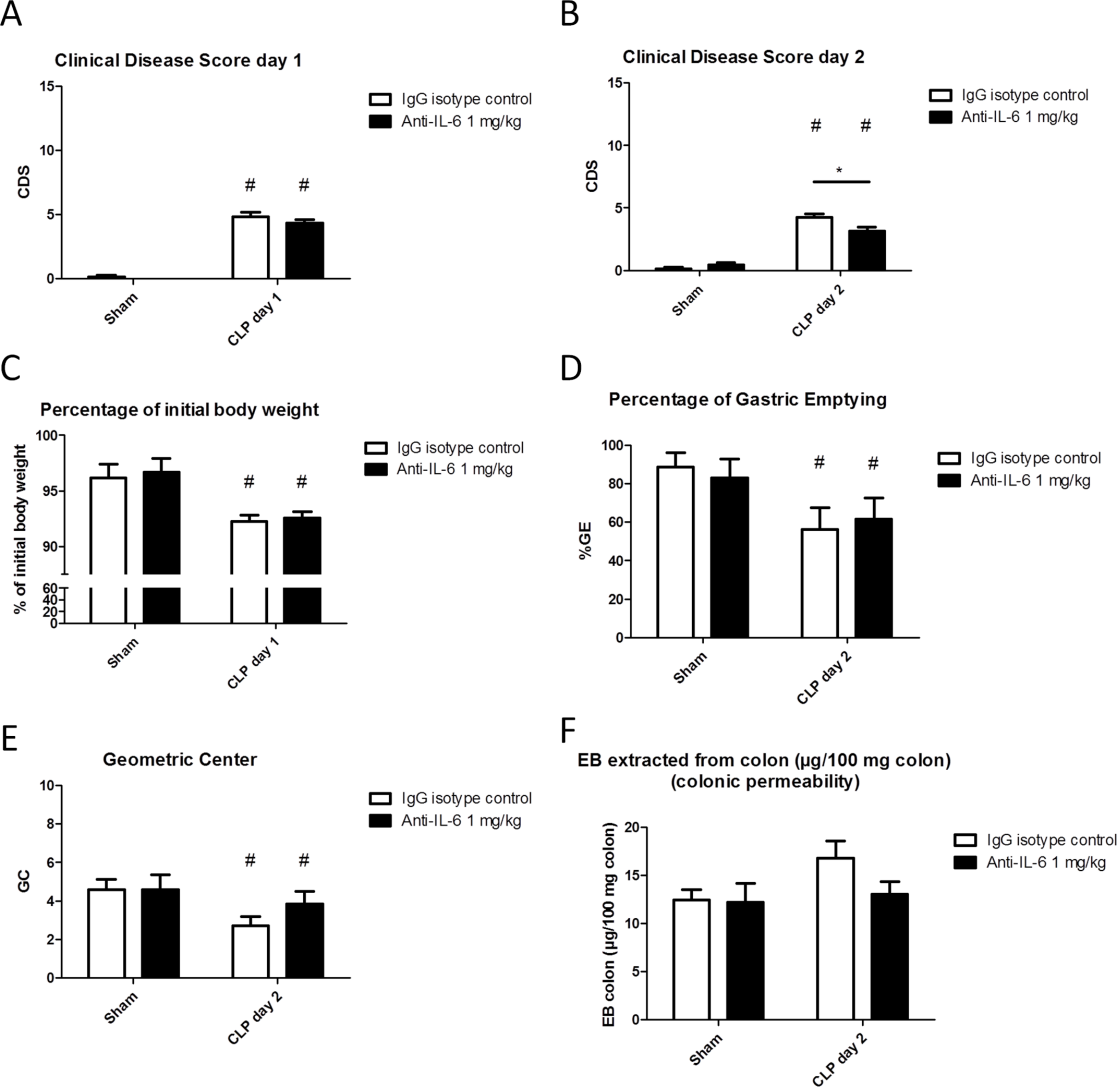
**Effect of** ***curative*** **treatment with anti-IL-6 antibodies on sepsis-induced clinical signs of disease 24h (A) and 48h (B) following CLP or sham-surgery, percentage of weight loss at day 1 (C), percentage of gastric emptying (D), geometric center of GI transit (E) and colonic permeability as measured by the *Evans blue* method (F).** Two-way ANOVA followed by One-way ANOVA and SNK *post-hoc* testing when appropriate, or its non-parametric equivalent for ordinal data; n = 7–10/group for A, B and C; n = 9–12/group for D; *p ≤ 0.05, # significant effect of CLP. CDS: clinical disease score; CLP: caecal ligation and puncture; GC: geometric center; %GE: percentage of gastric emptying; GI: gastrointestinal.

At day 1 and 2 following the CLP procedure, sepsis significantly increased the CDS. Curative treatment with anti-IL-6, 48h after the CLP procedure, had a favorable effect on the CDS at the moment of sacrifice (Fig 5B). The body weight of septic animals was significantly decreased in comparison to their non-septic counterparts (Fig 5C). Sham-animals treated with anti-IL-6 antibodies in a curative setup did not display clinical signs of disease. No difference in survival was noted between the anti-IL-6 treated and isotype-treated septic animal group as two animals in both groups had succumbed to the procedure prior to sacrifice.

Sepsis significantly reduced gastrointestinal transit as shown by a reduced intestinal geometric center (Fig 5E). Curative treatment with anti-IL-6 antibodies did no longer result in a significant amelioration of the GI transit of CLP-treated animals.

Similar to our findings in the preventive setup, serum and colonic proinflammatory cytokine levels were significantly augmented following CLP in the curative set-up. Curative treatment with anti-IL-6 24h following the CLP-procedure significantly decreased serum levels of IL-6 and IL-10, as well as colonic levels of IL-6 and TNF-α. Serum levels of other cytokines however were not significantly altered by the curative injection with anti-IL-6 antibodies. The same was true for the colonic supernatants. Results at the colonic level were for the greater part confirmed by RT-PCR at the mRNA level (Table 6). Concerning the assessment of colonic permeability, the significant beneficial effect of the antibodies that could be observed in the preventive protocols was lost in the curative one (Fig 5F).

**Table 6:** Cytokine levels in serum (A) and supernatants of homogenized colons (B) measured by CBA, and determined by RT-PCR in colon (C) during the *curative* set-up (administration of anti-IL-6 antibodies 24h following the CLP- or sham-procedure).

	sham + IgG isotype	sham + anti-IL-6	CLP d2 + IgG isotype	CLP d2 + anti-IL-6
**A. Serum cytokines** (pg/mL)				
IL-6	23.46 ± 4.46	4.14 ± 1.44^2^	855.20 ± 462.37^1^	54.7 ± 5.54^1^^,^^2^
TNF-α	13.85 ± 1.12	19.48 ± 7.19	279.15 ± 133.12^#^	62.09 ± 10.89^#^
IL-10	3.36 ± 0.56	3.27 ± 0.72^§^	11.74 ± 3.56^#^	3.90 ± 0.78^#^^,^^§^
IL-1β	1.35 ± 0.50	6.86 ± 5.70	9.23 ± 6.36	3.17 ± 1.11
IL-17A	2.14 ± 0.52	2.49 ± 0.35	4.71 ± 2.02^#^	5.88 ± 1.29^#^
IL-1α	1.90 ± 0.50	2.08 ± 0.61	10.99 ± 5.51^#^	7.46 ± 2.60^#^
**B. Colon cytokines** (pg/100 mg colon tissue)				
IL-6	4.27 ± 1.20	3.28 ± 0.89	432.19 ± 151.88^a^^,^^b^	68.04 ± 7.97^c^
TNF-α	6.21 ± 0.69	5.36 ± 1.43^3^	31.79 ± 6.54^#^	18.38 ± 3.63^#^^,^^3^
IL-10	<L/D	<L/D	<L/D	<L/D
IL-1β	2.01 ± 0.66	3.99 ± 1.68	39.54 ± 20.25^#^	10.31 ± 2.05^#^
IL-17A	<L/D	<L/D	2.58 ± 1.09	1.17 ± 0.21
IL-1α	2.10 ± 0.19	1.70 ± 0.18	16.96 ± 6.51^#^	7.72 ± 2.64^#^
**C. mRNA colonic tissue**				
IL-6	1.36 ± 0.38	1.27 ± 0.19^§^	117.79 ± 43.89^#^	25.50 ± 5.54^#^^,^^§^
TNF-α	1.28 ± 0.32	1.46 ± 0.24	7.31 ± 1.30^#^	5.20 ± 1.08^#^
IL-10	1.10 ± 0.17	1.65 ± 0.17	4.74 ± 0.51^#^	4.13 ± 0.50^#^
IL-1β	1.30 ± 0.33	1.00 ± 0.15	8.91 ± 2.06^#^	5.89 ± 0.99^#^
IL-1α	1.99 ± 0.71	1.51 ± 0.25^§^	9.61 ± 2.54^#^	3.28 ± 0.85^#^^§^

Two-way ANOVA followed by one-way ANOVA and SNK post-hoc testing when appropriate.

# significant effect of CLP

§ significant effect of anti-IL-6

For the SNK post-hoc testing

^a^ <0.05 compared to sham + IgG isotype

^b^ <0.05 compared to sham + anti-IL-6

^c^ <0.05 compared to CLP d2 + IgG isotype.

^1^ p = 0.07 for the effect of CLP

^2^ p = 0.06 for the effect of anti-IL-6

^3^ p = 0.07 for the effect of anti-IL-6.

When sham concentrations were below the theoretical limit of detection, the unpaired Student’s t-test was subsequently applied to compare the CLP d2 with the CLP d2 + anti-IL-6 group. For the PCR-results, data are expressed as relative expression (2^-ΔΔCT^ method) and the *sham + vehicle* group was chosen as calibrator. Data are presented as mean ± SEM. N = 10–12 animals per group.

<L/D, below the limit of detection; CBA, cytometric bead array; IL, interleukin; TNF, tumor necrosis factor.

The mRNA-levels of claudin-1, occludin and E-cadherin were significantly higher during sepsis, and septic animals curatively treated with anti-IL-6 did not display this increase in mRNA levels (Table 4). Furthermore, the mRNA level of desmoglein-2 and zonulin-1 was also significantly lower in curatively treated animals in comparison to their non-treated septic counterparts. Administering anti-IL-6 had no influence on the outcome of hemocultures or MLN cultures (100% in IgG isotype control as well as anti-IL-6 treated CLP-animals) (Table 3C*)*.

## Discussion

In the current study, we provide evidence for the beneficial effects of preventive treatment with antibodies against interleukin-6 on gastrointestinal motility disturbances, systemic and colonic inflammation and colonic permeability induced by polymicrobial abdominal sepsis. Sepsis was induced by caecal ligation and puncture, one of the most commonly used animal models in sepsis research. Increased secretion of pro- as well as anti-inflammatory cytokines was observed systemically in serum samples as well as locally in the colon. Preventive administration of anti-IL-6 antibodies in septic mice resulted in a significant drop of IL-6 and IL-10 levels in serum, as well as of the TNF-α concentration in the colon. Colonic IL-1α and IL-1β levels in septic animals were also increased during sepsis at the protein and mRNA level, and others showed that IL-1 signaling in enteric glia cells was required for the development of ileus [38], and that increased IL-1β levels in the gut muscularis coincided with a delay in intestinal transit in a mouse model of postoperative ileus [39]. The clinical disease score was significantly lower 24 h after the procedure in animals that preventively received anti IL-6 antibodies compared to vehicle-treated mice, and the impaired GI transit, as well as the colonic permeability, were significantly improved. We confirmed a significant rise in claudin-1 levels in colonic levels, a finding that was also already communicated by others and which probably represents an attempted repair response [40,41]. Claudin-1 levels normalized following treatment with anti-IL-6. It should be kept in mind that we utilized whole organ specimens to assess mRNA levels, which could theoretically be influenced by infiltration of leukocytes and villus blunting due to cell death. These features however were very limited at day 2 post-CLP in our animal model. Our results therefore show that targeting IL-6 prevented the occurrence of impaired GI transit, local and systemic inflammation and increased permeability induced by the CLP-procedure

Interestingly, these beneficial effects of anti-IL-6 were even more pronounced when the injection with the antibodies was repeated once 24h following the CLP- or sham-procedure. Interleukin-6 levels in serum and colon were normalized to control values, with a concomitant significant improvement in GI transit and colonic barrier function, and a significant improvement in survival by day 2 post-CLP. However, we were unable to detect a decrease in the number of positive bacterial cultures from blood and MLN, believed to represent an important clinical outcome parameter. Of note, the CLP-model in itself is a peritonitis model, and a major source of bacteria originates not only from the lumen of the GI tract, but directly from the peritoneum. This presumably explains the discrepancy between the normalized colonic permeability, and consistent positive outcome of the cultures. Finally, in the human clinical setting, the outcome of blood cultures not always correlates well with the severity of sepsis.

It should be noted that the favorable effects of antibodies to IL-6 in the preventive setup on colon permeability could also be due to a concomitant effect of the antibodies on colonic TNF-α levels, as these were also significantly decreased. Many have shown that TNF-α has direct deteriorating effects on several epithelial barrier layers [41–45], therefore making it difficult to disentangle the direct (IL-6) versus indirect (via TNF-α) effects of anti-IL-6 on colonic permeability,.

This is in sharp contrast to the set-up in which anti-IL-6 administration was repeated 24h post-CLP, in which a reduction of colonic IL-6 levels compared to those observed in control animals actually resulted in an increase in TNF-α levels. This might be due to the fact that IL-6 also exerts anti-inflammatory effects on TNF-α via the release of soluble TNF-receptors [46], an effect which thus is now lost. Furthermore, we and others have also observed this phenomenon in preceding research [22,47,48], but also others have demonstrated an increased TNF-α serum level in mice treated with anti-IL-6 antibodies [49] or in IL-6 knock-out mice [50], confirming that IL-6 is involved in the regulation of TNF-α levels. Besides, the anti-inflammatory effect of IL-6 is also reflected by the increased serum-levels of IL-10 in isotype-treated septic animals that dropped concomitantly with IL-6 following treatment with the antibodies.

In the animals that were treated with antibodies to IL-6 in a curative fashion, the beneficial effects were clearly less pronounced. The effects on GI transit and colonic permeability even failed to reach significance. Septic animals curatively treated with anti-IL-6 however still demonstrated significantly lower expression levels of IL-6, IL-10 and IL-1α, systemically and/or locally in the colon. Our results therefore suggest that IL-6 plays a major role in the initiation of sepsis-related alterations in GI motility, permeability and inflammation, but loses its potency partially once sepsis-induced alterations are full-blown. This indicates that the earlier the treatment with anti-IL-6 starts, the more pronounced the beneficial outcome on inflammation and GI function will be, although our results also support the fact that repeated administration is beneficial and even mandatory to maintain the beneficial effects on permeability, inflammation and motility. Our findings hence also indicate that a specific anti-cytokine strategy may offer therapeutic possibilities as part of the medical armamentarium in the early treatment of the patient developing a septic state. Furthermore, in our study a proinflammatory septic state was characterized by increased levels of several proinflammatory cytokines in the serum and colon, suggesting that other targets of anti-cytokine strategy might be worth exploring.

It has been repeatedly suggested that the optimal choice of dosage and time of administration of anti-IL-6 is pivotal in order to obtain beneficial effects during sepsis. Riedemann and colleagues demonstrated that a dosage of 1.33 mg/kg of anti-IL-6 injected i.v. at the start of CLP significantly increased survival, whereas a lower (0.33 mg/kg) or higher dosage (2.66 mg/kg) did not. Delayed infusion (4h post-CLP) resulted in a modest but not significant increase in survival when compared to animals treated with an irrelevant IgG [29]. In another murine CLP-model, therapy targeting IL-6 failed to improve survival whereas early administration of broad spectrum antibiotics was successful [28]. It should, however, be noted that these authors utilized a very severe form of CLP, which nearly always results in 100% mortality within 72h [51], and IL-6 levels were substantially higher in the latter study when compared to the former one: the average IL-6 serum level for the untreated septic animals was >14 000 pg/mL versus 7500 pg/mL respectively. Combined, these results indicate that the severity of sepsis, as deduced from the levels of IL-6, and the time of initiation as well as the maintenance treatment of anti-cytokine strategies will play a pivotal role in the therapeutic outcome. As human IL-6 levels are indicative of chances of survival during sepsis [52,53], a patient-centered decision on whether or not to administer antibodies to IL-6 is mandatory based upon a broader patient’s immunological profile. As single biomarkers such as CD14 and procalcitonin, despite being excellent diagnostic tools, often fail to predict clinical outcome, profiling patients using a combination of these markers, with the inclusion of IL-6, might represent an attractive approach [2,54].

Remarkably, the number of positive cultures from blood from septic mice did not decrease significantly following preventive treatment with anti-IL-6, and no effect of anti-IL-6 was seen at all on the MLN cultures. This is in contrast to our previous findings in which septic mice were treated with the alpha7 nicotinic acetylcholine receptor agonist GTS-21 [22]. By binding onto the alpha7 nicotinic acetylcholine receptor, GTS-21 prevents macrophages from secreting proinflammatory cytokines such as IL-6 and we showed that GTS-21 treatment normalized colonic permeability and significantly reduced the number of positive blood and MLN cultures from septic mice and normalized colonic and serum Il-6 levels. GTS-21 can, however, also bind onto the alpha4beta2 receptor on peritoneal macrophages, and stimulation of this specific receptor will induce the phagocytosis of bacteria [55]. These findings together with our current results suggest that the mere targeting of IL-6, despite resulting in a normalized intestinal permeability, probably does not suffice to halt bacterial translocation, especially since we were able to completely reduce serum and colonic IL-6 levels to those measured in control animals. The role of IL-6 in permeability has been established extensively in *in vitro* experiments, where it was shown that IL-6 increased endothelial permeability [12] and it decreased membrane integrity in an enterocyte cell culture model [14]. Moreover, IL-6 increased claudin-2 expression, which actually decreases the intestinal tight junction barrier [56].

Some limitations of this study and considerations must be taken into account: a non-lethal CLP-model was chosen, and thus we refer the interested reader to several other manuscripts that tackle the clinical endpoint of survival *per se* [25,28,29]. Furthermore, IL-6 treated animals were not completely protected from systemic spread of invading pathogens, as cultures from blood and MLN still yielded positive results following treatment with anti-IL-6.

In summary, our data confirm a time-dependent beneficial effect of blocking IL-6 via the administration of specific blocking antibodies in a mouse model of polymicrobial abdominal sepsis. Beneficial effects were observed on gastrointestinal motility, inflammation and permeability, with effects being predominantly pronounced when the antibodies were administered immediately prior to the induction of instead of during the course of the sepsis. This demonstrates that IL-6 is not only an important initiator of inflammation, but also affects GI motility and permeability. Whether IL-6 is the main direct acting mediator, or whether it exerts its effects indirectly remains the subject of further research.

Anti-cytokine strategies hence seem to offer interesting therapeutic possibilities, given the fact that they are administered in an appropriate dosage, at the suitable moment, including a maintenance regimen if necessary, and in a carefully selected patient along with other conventional therapies.

## Supporting Information

S1 Fig Hematoxylin-eosin staining of proximal colon. Representative haematoxylin-eosin staining in vehicle-treated sham (A) and CLP-animals (C), and animals preventively treated with antibodies to IL-6 treated (sham (B) and CLP-animals (D)). 200x magnification.
(TIF)

S1 Table Research literature investigating the effect of anti-interleukin-6 antibodies in mouse models of sepsis and/or (septic) shock. (DOCX)

S2 Table Taqman primers used in the aforementioned protocols. (DOCX)

## Abbreviations

%GE: percentage of gastric emptying
anti-IL-6: anti-mouse interleukin-6 antibodies
CDS: clinical disease score
CLP: caecal ligation and puncture
EB: Evans blue
GC: geometric center
GI: gastrointestinal
IgG: immunoglobulin G
IL: interleukin
i.p.: intraperitoneally
KO: knock-out
LPS: lipopolysaccharide
MLN: mesenteric lymph node
PBS: phosphate-buffered saline
s.c.: subcutaneously
sIL-6R: soluble interleukin-6 receptor
TNF: tumor necrosis factor
