# Supplementary figures and images for: Beneficial Effects of Anti-Interleukin-6 Antibodies on Impaired Gastrointestinal Motility, Inflammation and Increased Colonic Permeability in a Murine Model of Sepsis Are Most Pronounced When Administered in a Preventive Setup
Source: PLoS One. 2016 Apr 4;11(4):e0152914. doi: 10.1371/journal.pone.0152914 (PMC4820138; doi:10.1371/journal.pone.0152914)

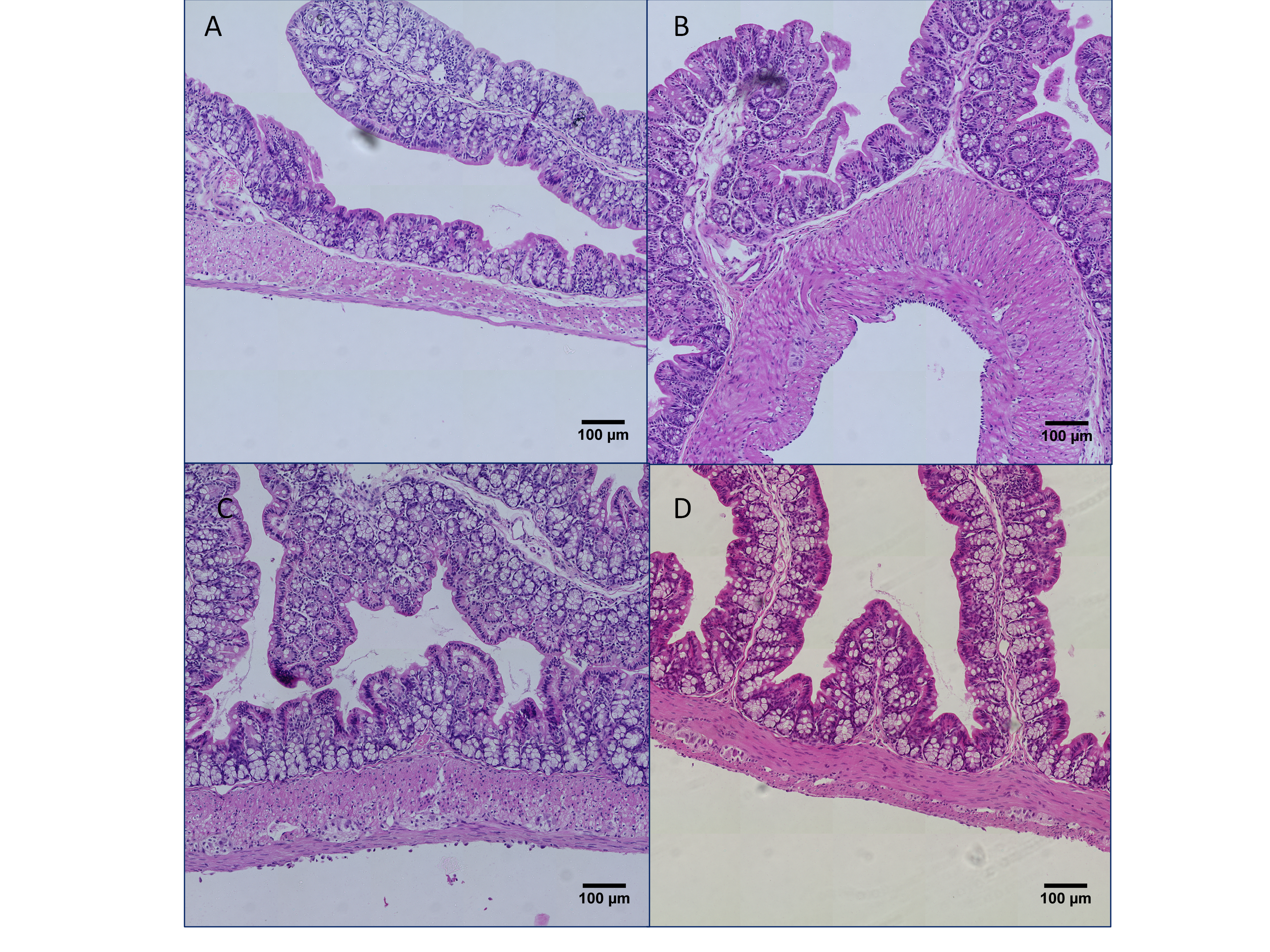

Supplement: S1 Fig — Representative haematoxylin-eosin staining in vehicle-treated sham (A) and CLP-animals (C), and animals preventively treated with antibodies to IL-6 treated (sham (B) and CLP-animals (D)). 200x magnification. (TIF) [file pone.0152914.s001.tif]
