# Supplementary material for: Beneficial Effects of Anti-Interleukin-6 Antibodies on Impaired Gastrointestinal Motility, Inflammation and Increased Colonic Permeability in a Murine Model of Sepsis Are Most Pronounced When Administered in a Preventive Setup
Source: PLoS One. 2016 Apr 4;11(4):e0152914. doi: 10.1371/journal.pone.0152914 (PMC4820138; doi:10.1371/journal.pone.0152914)
Supplement: S1 Table — (DOCX) [file pone.0152914.s002.docx]

| **Authors** | **Animal model** | **Intervention** | **Observed effects** |
| --- | --- | --- | --- |
| Libert et al, 1992 (48) | Injection with LPS | Antimurine IL-6 antibody | Anti-IL-6 protected against LPS, but not against the highest dosage. |
| Gennari et al, 1994 (27) | Bacterial gavage and burn injury | Antimurine IL-6, IgG or placebo 1h before insult | Increased survival following anti-IL-6 therapy and a decrease in bacterial translocation. |
| Gennari et al, 1995 (26) | Bacterial gavage and thermal injury | Antimurine IL-6, IgG or placebo 2/4/8h after insult | Anti-IL-6 enhanced clearance of bacteria when given 2h post-insult. |
| Wang et al, 2001 (30) | Caecal ligation and puncture | IL-6 WT and KO mice | Intestinal permeability was increased in septic IL-6 WT mice, but not in IL-6 KO mice |
| Pallua et al, 2003 (25) | Contact burn followed by LPS injection | Anti-IL-6 and/or anti-IL-6R antibody | Both compounds effectively prevented systemic inflammation, effect was more pronounced when administered after LPS injection. |
| Riedemann et al, 2003 (29) | Caecal ligation and puncture | Anti-IL-6 (different dosages) immediately or 4h after CLP | Improved survival in anti-IL-6 treated mice; highest dosage of anti-IL-6 increased mortality; delayed infusion yielded no survival benefit. |
| Yang et al, 2003 (15) | Hemorrhagic shock and resuscitation | WT mice and IL-6-KO mice | IL-6 was necessary to induce gut hyperpermeability and dysfunction. |
| Vyas et al, 2005 (28) | Caecal ligation and puncture | Early antibiotics or anti-IL-6 based upon serum IL-6 levels | Early antibiotic treatment improved outcome in mice predicted to die, no effect of anti-IL-6 on survival. |
| Mees et al, 2009 (49) | Hemorrhagic shock and caecal ligation and puncture | Blocking of *trans*-signalling with gp130Fc | Treatment with gp130Fc significantly reduced IL-6 levels, but survival was unaffected. |
| Barkhausen et al, 2011 (23) | Caecal ligation and puncture | sgp130Fc (inhibits IL-6 *trans*-signalling) or anti-IL-6, 24h before or 24h after CLP | Pretreatment with sgp130Fc ameliorated survival, whereas anti-IL-6 did not. |
| Mostafa Anower et al, 2011 (24) | Caecal ligation and puncture | Pretreatment with siRNA | Pretreatment with siRNA induced extended survival. |
