# Supplementary material for: Beneficial Effects of Anti-Interleukin-6 Antibodies on Impaired Gastrointestinal Motility, Inflammation and Increased Colonic Permeability in a Murine Model of Sepsis Are Most Pronounced When Administered in a Preventive Setup
Source: PLoS One. 2016 Apr 4;11(4):e0152914. doi: 10.1371/journal.pone.0152914 (PMC4820138; doi:10.1371/journal.pone.0152914)
Supplement: S2 Table — (DOCX) [file pone.0152914.s003.docx]

| ***Protein*** | ***Gene id*** |
| --- | --- |
| Interleukin-6 | 16193 - Mm00446190_m1 |
| Tumor Necrosis Factor-α | 21926 – Mm00443258_m1 |
| Interleukin-10 | 16153 - Mm00439614_m1 |
| Interleukin-17 | 16171 - Mm00439618_m1 |
| Interleukin-4 | 16189 - Mm00445259_m1 |
| Interleukin-1β | 16176 – Mm00434228_m1 |
| Interferon-γ | 15978 - Mm01168134_m1 |
| GAPDH | 14433 - Mm99999915_g1 |
| β-actin | 11461 - Mm00607939_s1 |
| 18S ribosomal RNA | 19791 – Mm03928990_g1 |
| Occludin | 18260 - Mm00500912_1 |
| Claudin-1 | 12737 - Mm00516701_m1 |
| Desmoglein-2 | 13511 - Mm00514608_m1 |
| E-cadherin | 12550 – Mm01247357_m1 |
| Zonulin-1 | 21872 - Mm00493699_m1 |
